# Integrated wall stress: a new methodological approach to assess ventricular workload and myocardial contractile reserve

**DOI:** 10.1186/1479-5876-11-183

**Published:** 2013-08-07

**Authors:** Hailong Dong, Heather Mosca, Erhe Gao, Robert E Akins, Samuel S Gidding, Takeshi Tsuda

**Affiliations:** 1 Nemours Cardiac Center and Nemours Biomedical Research, Alfred I. duPont Hospital for Children, 1600 Rockland Rd., Wilmington, DE 19103, USA; 2 Nemours Cardiac Center, Alfred I. duPont Hospital for Children, 1600 Rockland Rd., Wilmington, DE 19103, USA; 3 Center for Translational Medicine, Temple University, Philadelphia, PA 19140, USA; 4 Current address: Department of Anesthesiology, Xijing Hospital, Fourth Military Medical University, Xi’an, People’s Republic of China

**Keywords:** Wall stress, Ventricular workload, Myocardial contractile reserve, Ventricular remodeling

## Abstract

**Background:**

Wall stress is a useful concept to understand the progression of ventricular remodeling. We measured cumulative LV wall stress throughout the cardiac cycle over unit time and tested whether this “integrated wall stress (IWS)” would provide a reliable marker of total ventricular workload.

**Methods and results:**

We applied IWS to mice after experimental myocardial infarction (MI) and sham-operated mice, both at rest and under dobutamine stimulation. Small infarcts were created so as not to cause subsequent overt hemodynamic decompensation. IWS was calculated over one minute through simultaneous measurement of LV internal diameter and wall thickness by echocardiography and LV pressure by LV catheterization. At rest, the MI group showed concentric LV hypertrophy pattern with preserved LV cavity size, LV systolic function, and IWS comparable with the sham group. Dobutamine stimulation induced a dose-dependent increase in IWS in MI mice, but not in sham mice; MI mice mainly increased heart rate, whereas sham mice increased LV systolic and diastolic function. IWS showed good correlation with a product of peak-systolic wall stress and heart rate. We postulate that this increase in IWS in post**-**MI mice represents limited myocardial contractile reserve.

**Conclusion:**

We hereby propose that IWS provides a useful estimate of total ventricular workload in the mouse model and that increased IWS indicates limited LV myocardial contractile reserve.

## Introduction

Ventricular remodeling is a chronic progressive pathological process that results in heart failure after myocardial infarction (MI) or persistent unrelieved biomechanical overload [1,2]. Persistent and unrelieved biomechanical overload in combination with activation of inflammatory mediators and neurohormones is thought to be responsible for progressive ventricular remodeling after MI [3,4], but studies to investigate specific mechanisms in animals are hampered by the difficulty involved in quantifying biomechanical workload *in vivo*. The magnitude of ventricular remodeling advances in line with progressive ventricular geometric changes including myocardial hypertrophy and chamber dilatation with accompanying functional deterioration [1,2]. Previously, we proposed that post-ischemic ventricular remodeling is a pathological spectrum ranging from benign myocardial hypertrophy to progressive heart failure in the mouse model in which the prognosis is primarily determined by the magnitude of residual hemodynamic effects [5]. However, there has been no optimum quantitative measurement of ventricular workload as a contributory indicator of ventricular remodeling other than wall stress theory to explain how ventricular dilatation and hypertrophy develop after loss of viable working myocardium [6,7].

The concept of ventricular wall stress was introduced by Strauer et al. as a primary determinant of myocardial oxygen demand [8]. They indicated that overall myocardial energy demand depends upon intramyocardial wall tension, inotropic state of the myocardium, and heart rate. Wall stress theory is commonly introduced to explain development of concentric hypertrophy in chronic pressure overload and progressive ventricular dilatation in the failing heart. One study argued that peak-systolic wall stress increased as LV function worsened in a chronic volume overloaded status [9], and another suggested that peak-systolic wall stress closely reflected LV functional reserve during exercise [10]. However, the effect of heart rate or myocardial contractility was not considered in either study. Heart rate has been shown to be one of several important factors contributing to myocardial oxygen consumption [11].

Herein, we introduce a novel concept of “integrated wall stress (IWS)” to assess its significance as a marker of total ventricular workload and to validate its physiological relevance in the mouse model. The concept of continuous LV wall stress measurement was reported previously, but authors did not address the overall effects of changing wall stress during the cardiac cycle on the working myocardium [12]. We have defined IWS as cumulative wall stress over unit time: IWS was obtained by integrating continuous wall stress curve by accumulating wall stress values at millisecond sampling intervals over 1 min. By calculating IWS, we were able to incorporate the effects of not only systolic wall stress, but also of heart rate and inotropic status of the myocardium. These data were analyzed against conventional hemodynamic parameters in animals with and without MI in conjunction with incremental dobutamine stress. We hypothesize that unchanged IWS represents stable ventricular myocardial contractile reserve and that increase in IWS implies an early sign of mismatch between myocardial reserve and workload imposed on ventricular myocardium.

## Methods

### Animals

A total of twenty (20) male C57BL/6 mice were used according to an IACUC-approved protocol. Ten mice were randomly assigned to an MI group and the other 10 to a sham group. The investigation conformed with the *Guide for the Care and Use of Laboratory Animals* published by the US National Institute of Health (NIH Publication No. 85-23, revised 1996).

### Experimental MI by coronary artery ligation

Experimental MI was induced as described by the authors in detail elsewhere [5,13]. Briefly, under isoflurane anesthesia (2%), the heart was exposed via small left thoracotomy, and suture ligation was placed at the distal one-third of the left anterior descending coronary artery (LAD) with a 6.0 silk suture. With this procedure, approximately 35% of LV myocardium was subjected to permanent ischemia, but only 14% became infarcted at both 24 and 72 hours after MI [5]. Because of the relatively small size of infarction, MI mice did not develop hemodynamic deterioration but marked compensatory LV hypertrophy in the non-ischemic myocardium 1 week after MI [5]. For post-operative pain management, meloxicam 5 mg/kg was given subcutaneously after the experimental MI before transferring back to vivarium. All mice survived for 7 wks after the procedure.

### Echocardiography

Echocardiography was performed at baseline and weekly after LAD ligation using a 30 MHz transducer (Vevo770, VisualSonics, Toronto, Canada) under isoflurane anesthesia (1.5%). Once the parasternal short-axis view of the LV was established by B-mode, M-mode tracings were recorded to measure left ventricular internal diameter in diastole (LVIDd), left ventricular posterior wall thickness in diastole (LVPWd), and left ventricular internal diameter in systole (LVIDs). Calculated values are fractional shortening (%FS) = {(LVIDd – LVIDs)/LVIDd} × 100, and the mean velocity of circumferential fiber shortening (%Vcf) = (LVIDd – LVIDs)/(LVIDd × ET) (ET: ejection time in sec).

### Hemodynamic assessment with echocardiogram

Seven weeks after experimental MI, the mice were anesthetized with intraperitoneal injection of ketamine (50 μg/g body weight) and xylazine (5 μg/g body weight). A 1.4 Fr Millar Mikro-tip Catheter Transducer (Model SPR-671, Millar Instruments, Houston, TX) was inserted through right common carotid artery cutdown into the LV cavity. The LV pressure signals were ported to the Vevo770 Ultrasound System in real time. Simultaneously, M-mode echocardiogram was obtained from parasternal short axis view. LV systolic pressure (SP) and LV end diastolic pressure (LVEDP) were measured. The maximum values of the first derivatives of the ascending (+) and descending (−) left ventricular pressure [(+)dP/dT_max_, (−)dP/dT_max_] were also obtained. LV pressure waveform input and pressure derivative (dP/dT) were superimposed on the electrocardiogram (ECG) trace in real time along with data regarding heart rate (HR) and body temperature (ºC).

### Dobutamine stress test

A PE-10 catheter (Becton Dickinson, Mountain View, CA) was inserted retrograde into the left jugular vein by cutdown for dobutamine infusion at the time of hemodynamic evaluation, as described elsewhere [14]. Dobutamine was infused at the rate of 5 and 10 μg/kg/min with microinfusion pump (KDS100, KD Scientific, Holliston, MA). Hemodynamic data were obtained after 3 minutes of dobutamine infusion at the specified rate.

### Continuous wall stress (CWS) curve and IWS

**Figure 1:**
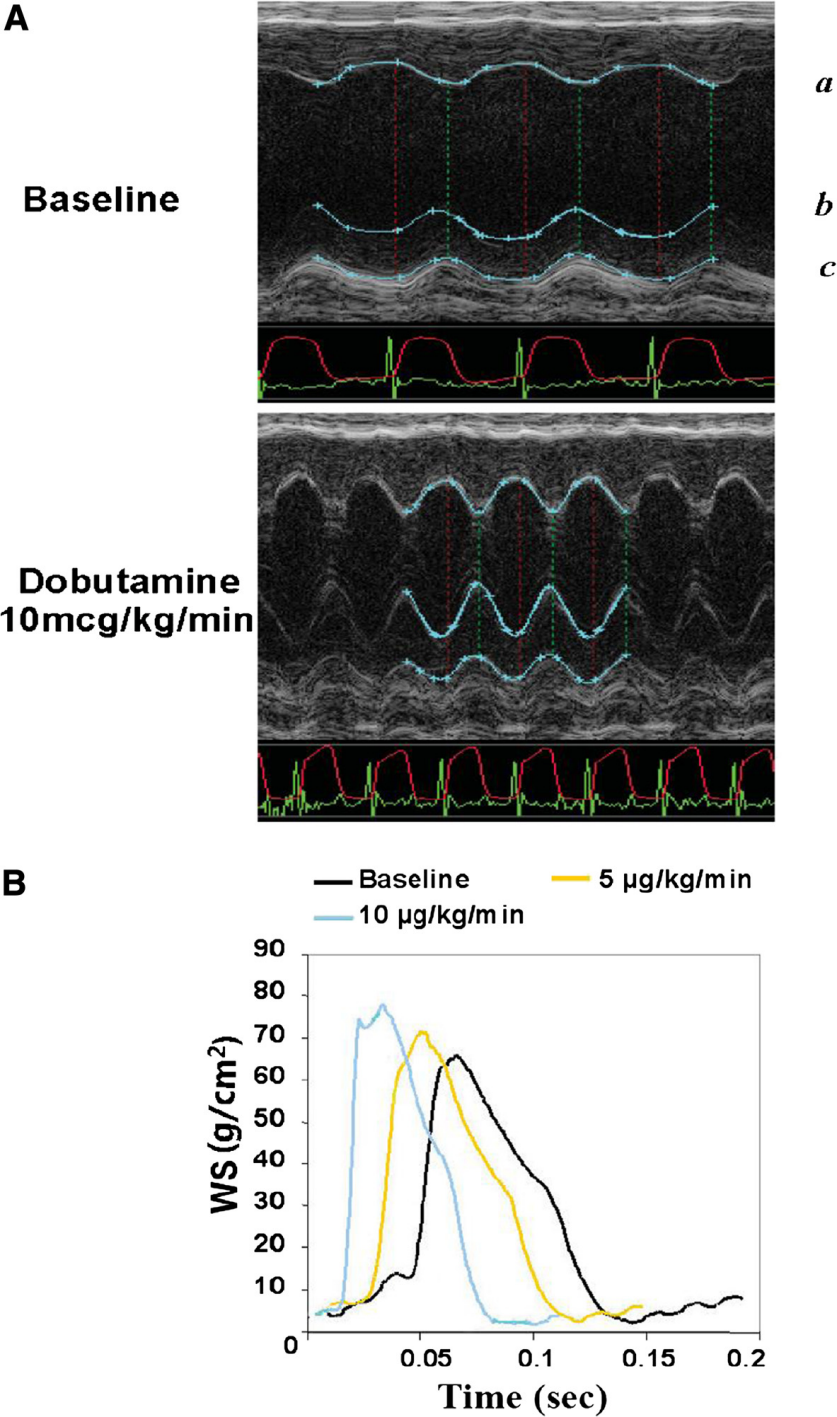
**Continuous wall stress measurement in the mouse model. A**. Simultaneous M-mode echocardiogram and LVP monitoring in obtaining continuous wall stress (CWS). The CWS curve was obtained from direct simultaneous measurement of LVID, LVPW, and LVP every 1 msec. Blue line outlines LV endocardial surfaces **(*a*** and ***b*)** and epicardial surface **(*c*)**. Red line and green line indicate electrocardiogram (ECG) and LVP measurement, respectively. When calculating wall stress, LVID and LVPW were used as inner diameter and wall thickness, respectively. **B**: CWS curve obtained from the formula (see text) with dobutamine stimulation (baseline, 5, and 10 μg/kg/min) in the normal control mouse. IWS was calculated as an area below CWS curve over unit time.

CWS curves were obtained via the simultaneous measurement of LVID and LVPW by M-mode echocardiogram and LV pressure by cardiac catheterization (Figure 1A). LV wall stress (σ) was calculated as follows [12,15]:

$$s_{m}={\mathrm{PR}}_{i}^{2}/\left[\left(R_{o}-R_{i}\right)\left(R_{o}+R_{i}\right)\right]={\mathrm{PR}}_{i}/2h\left(1+h/{2R}_{i}\right)$$

P = Pressure, R_*o*_ = Outer diameter, R_*i*_ = Inner diameter (LVID), h = Wall thickness (LVPW)

The IWS was calculated as an integration of continuous wall stress over unit time (60 sec or 60,000 msec), which was equivalent to the area under the continuous wall stress curve.

$$\mathrm{IWS}=\int \sigma_{m}\mathrm{dt}$$

### Anatomical and histological analyses to assess ventricular remodeling

After hemodynamic and IWS measurements were completed, the animal was euthanized and the heart was excised. The LV/body weight ratio was calculated as a gross indicator of cardiac hypertrophy. Then, non-infarct LV myocardium was collected for molecular and histological examinations by obtaining the LV myocardium proximal to the suture site. For histology, the myocardial tissue was fixed in 10% formalin/PBS and was embedded in paraffin. Hematoxylin and eosin (H&E) staining and Masson’s trichrome staining were performed to assess basic tissue architecture and degree of myocardial fibrosis, respectively.

### Real time reverse transcriptase polymerase chain reaction (qRT-PCR)

Total RNA was extracted from non-infarct region of the LV myocardium of MI hearts and the same anatomical region of sham hearts with TRIreagent (Applied Biosystems, Foster City, CA). After DNAse treatment (RNase-Free DNase Set, Qiagen Inc, Valencia, CA), mRNA was reverse-transcribed to synthesize first-strand cDNA (iScript cDNA Synthesis Kit, Bio-RAD Laboratories, Inc., Hercules, CA). For PCR amplification, SYBR Green 1 kits (Bio-RAD Laboratories) were used with validated primers (see Table 1). PCR was carried out in a MyiQ Single-Color Real-Time PCR Detection System (Bio-Rad Laboratories). Data were recorded and analyzed using MyiQ software with glyceraldehyde-3-phosphate dehydrogenase (GAPDH), which showed consistent expression across all our samples, as an internal control. The relative expression of each gene was determined as the cycle threshold (Ct) of the gene of interest relative to GAPDH by the following formula:

$$\Delta \mathrm{Ct}=2^{\left[\left(\mathrm{Ct}\;\mathrm{GAPDH}\right)-\left(\mathrm{Ct}\;\mathrm{gene}\;\mathrm{of}\;\mathrm{interest}\right)\right]}$$

**Table 1:** The real-time RT-PCR primers used in this study

**Target**	**Sequence (5′-3′)**	**Pro. Size**
**ANP**	AGGAGAAGATGCCGGTAGAAGA	67
	GCTTCCTCAGTCTGCTCACTCA	
**BNP**	TGCTTTGGGCACAAGATAGA	122
	AGACCCAGGCAGAGTCAGAA	
**Col I**	GCG AGT GCT GTG CTT TCT G	68
	TCC CTC GAC TCC TAC ATC TTC	
**Col III**	CCCAACCCAGAGATCCCATT	73
	GAAGCACAGGAGCAGGTGTAGA	
**GAPDH**	TGCACCACCAACTGCTTAGC	77
	GTGGTCATGAGCCCTTCCA	

### Statistical analysis

Descriptive statistics were calculated. Two-tailed, paired *t*-tests were used to compare the anatomical, hemodynamic, and molecular data between MI and Sham groups. Comparisons of the temporal parameters (blood pressure and echocardiographic data) of MI and sham hearts were performed by one-way analysis of variance (ANOVA) followed by the Student-Newman-Keuls post-hoc test to assess the significance of data values (*p* < 0.05 considered significant). All data are expressed as mean ± SEM unless otherwise noted.

## Results

### Measurement of CWS in the mouse model

The ages of mice were 7.80 ± 1.55 months old. Their body weights ranged from 25 to 35 g (30.13 ± 3.19 g). CWS was obtained via simultaneous measurement of LV pressure (P), LVID (2R_*i*_), and LVPW (h) (Figure 1A). WS was calculated every 1 msec to generate an essentially CWS curve, which appears quite similar to the curve obtained in the human study [12] and in the canine model [11]. With the incremental infusion of dobutamine, the CWS curve of one of the sham operated mice shifted to the left with decreased cycle length and increased peak value (Figure 1B). IWS was calculated by integrating the WS data over 60,000 samplings (= 1 min). IWS changes with dobutamine infusion are shown later.

### Gradual LV geometric changes after small MI

**Figure 2:**
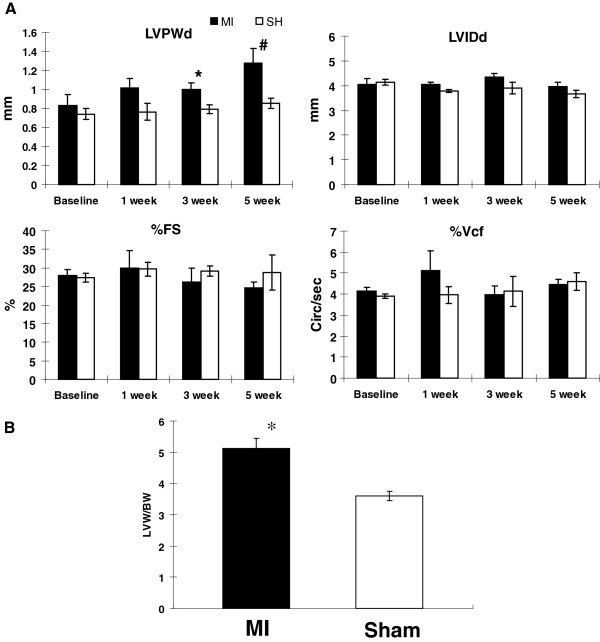
**Assessment of ventricular remodeling by echocardiography (A) and LVW/BW ratio. A**. Echocardiographic parameters at 1, 3, and 5 weeks after MI (n = 5) and sham groups (n = 5) under isoflurane anesthesia. A progressive increase in LVPWd was seen in MI group compared with sham, but there were no significant differences in LVIDd, %FS, or %Vcf between the two groups. * *p* < 0.05 vs. sham, # *p* < 0.05 vs. baseline. MI: myocardial infarction, SH: sham. **B**. LVW/BW ratio (mg/g) was significantly higher in MI group (n = 5) than in sham group (n = 5) (MI: 5.11 ± 0.32, sham 3.60 ± 0.35; *p* < 0.05), supporting the echocardiographic finding of LV hypertrophy in MI group. * *p* < 0.05 vs. sham.

To assess the efficacy of IWS, we examined echocardiographic and hemodynamic changes as well as IWS in post-MI and sham-operated hearts. There was no mortality following small MI throughout the 7 weeks of observation. Echocardiogram was obtained at 0, 1, 3, and 5 weeks after MI under isoflurane anesthesia to assess the temporal changes in LV dilatation [LVID(d)], LV hypertrophy [LVPW(d)], and systolic performance (%FS and %Vcf) in MI (n = 5) and sham (n = 5) groups (Figure 2A). Progressive ventricular hypertrophy was seen in the MI group as indicated by a continuous increase in LVPWd after MI. The LV cavity size remained relatively unchanged in both groups. This experimentally created small MI was restricted to the LV apex [5], and echocardiogram revealed that the majority of the LV appeared symmetrical, and LV myocardium including LV free wall and interventricular septum showed normal symmetric ventricular wall motion without segmental hypokinesia or dyskinesia. LV systolic performance presented by %FS and %Vcf was comparable between the two groups. These results indicate that the small MI procedure induced nearly concentric LV hypertrophy without LV chamber dilatation. The presence of ventricular hypertrophy in the MI group was also confirmed by LV (mg)/body (g) weight ratio, which was significantly higher than in the sham group (MI 5.11 ± 0.32 vs. sham 3.59 ± 0.16, *p* < 0.05; Figure 2B).

### Molecular and histological evidence of ventricular remodeling in the small MI model

**Figure 3:**
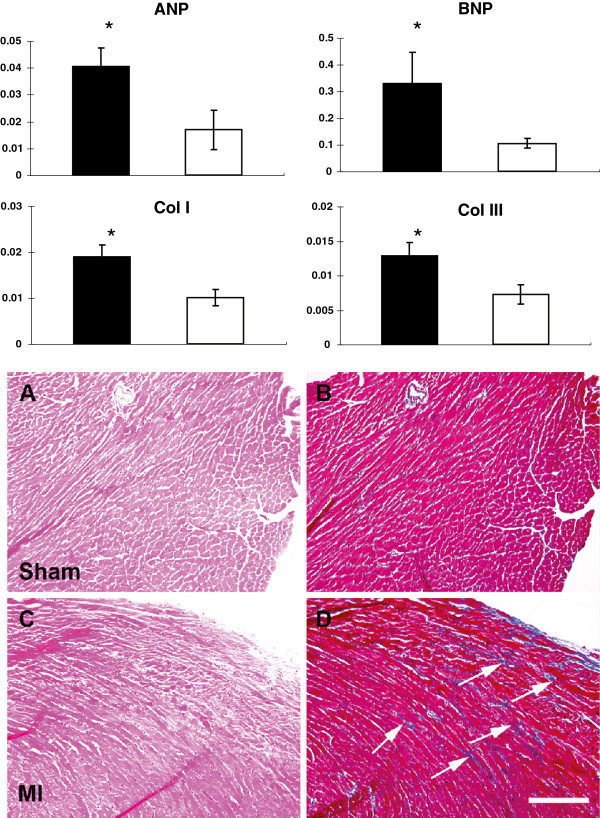
**Molecular expression and histological changes in non-ischemic myocardium in sham and MI mice. Upper**: The mRNA levels of ANP, BNP, and collagens type I and III in the non-ischemic myocardium of MI (n = 5) and sham (n = 5) groups by real-time RT-PCR to assess the molecular aspects of myocardial hypertrophy and fibrosis. ANP, BNP, and collagens type I and III were all significantly up-regulated in MI myocardium compared with sham myocardium. * *p* < 0.05 vs. sham. **Lower**: Myocardial fibrosis in the remote non-ischemic myocardium after MI. Histological assessment of remote non-ischemic myocardium with H&E (left column; **A** and **C**) and Masson’s Trichrome staining (right column; **B** and **D**. Collagen stains as blue color) in sham **(A**, **B)** and MI **(C**, **D)** hearts. In MI hearts, there is an increase deposition of collagen in the interstitial space (arrows) in the remote non-ischemic myocardium suggesting the development of myocardial fibrosis. Magnification bar = 100 μm.

This small MI model was previously reported by us as an experimental model to induce ventricular remodeling without initial hemodynamic deterioration [5]. After 7 weeks, a significant increase in the mRNA of hypertrophic markers, including ANP and BNP, was seen in the non-ischemic hypertrophied myocardium of the MI group, when compared with that of sham animals (Figure 3). In addition, mRNA levels of collagens type I and type III were also significantly elevated compared with that of the sham group, consistent with histological findings of increased interstitial fibrosis in the remote non-ischemic myocardium in the MI group (Figure 3). Molecular and histological findings of ventricular remodeling were seen in non-ischemic myocardium after small MI but without ventricular dilatation (Figure 2A).

### Echocardiographic and hemodynamic evaluation before and after dobutamine infusion

**Figure 4:**
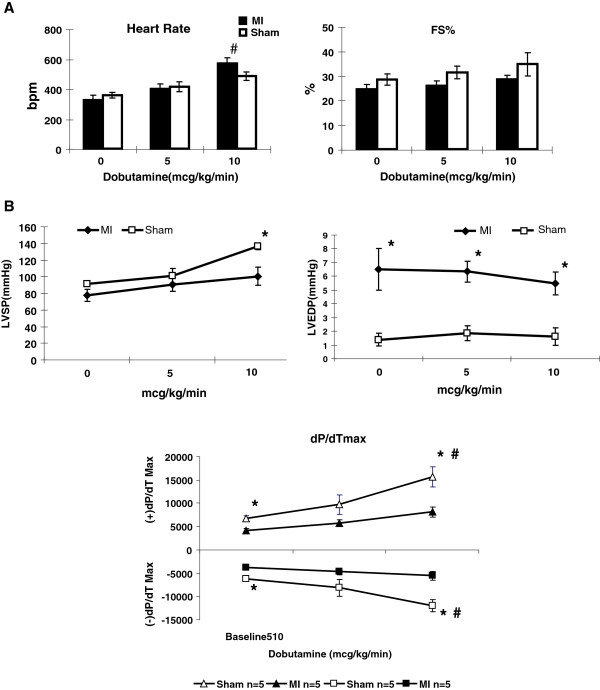
**Echocardiographic and hemodynamic changes with dubutamine stimulation after small MI. A**: Echocardiographic findings at 7 weeks after MI with dobutamine stimulation (baseline, 5, and 10 μg/kg/min for MI [n = 5] and sham groups [n = 5]) under xylazine/ketamine anesthesia. Significant heart rate increases were noted in MI group at 10 μg/kg/min, but not in sham group. %FS did not significantly change in response to dobutamine in either group, although there is a mild dose-dependent positive trend in sham mice. ^#^*p* < 0.05 vs. baseline. **B:** Hemodynamic data by cardiac catheterization at 7 weeks in MI (n = 5) and sham group (n = 5) with dobutamine stimulation (baseline, 5 μg/kg/min, and 10 μg/kg/min). LVSP was comparable between MI and sham at the baseline but became significantly higher in sham than in MI with maximum dobutamine stimulation. LVEDP was significantly higher in MI than in sham group at the baseline, and these values did not significantly change with dobutamine stimulation. Maximum response in (+)dP/dT_max_ and (−)dP/dT_max_ by dobutamine infusion from baseline were shown. Even at the baseline, sham hearts showed better systolic and diastolic LV function than MI hearts. In addition, sham hearts had better response to dobutamine stimulation for both systolic and diastolic function. * *p* < 0.05 vs. sham; # *p* < 0.05 vs. baseline.

At 7 weeks after MI, echocardiography and cardiac catheterization were performed simultaneously at rest and with continuous intravenous infusion of dobutamine (first 5 and then 10 μg/kg/min) under ketamine/xylazine anesthesia (Figure 4). Heart rate increase with dobutamine stimulation was more prominent in the MI group than in sham group. %FS by echocardiogram showed positive response by dobutamine, more in the sham group than in the MI group, although the increase was not statistically significant (Figure 4A). In hemodynamic evaluation, LVSP increased significantly with 10 μg/kg/min of dobutamine infusion in sham, whereas the increase was only modest in the MI group (Figure 4B). LVEDP was notably higher in MI than in sham, but the value did not change throughout dobutamine stimulation in either group. At the baseline, the absolute values of both (+)dP/dT_max_ and (−)dP/dT_max_ were significantly higher in the sham group than in the MI group (6820 ± 573 vs. 4115 ± 485 [*p* < 0.05], and –6144 ± 614 vs. –3824 ± 540 [*p* < 0.05], respectively); further difference was observed with the 10 μg/kg/min of dobutamine infusion (15681 ± 2128 vs. 8118 ± 1144 [*p* < 0.05] and –11986 ± 1257 vs. –5542 ± 920 [*p* < 0.05], respectively), suggesting that both systolic and diastolic ventricular reserve capacities were significantly diminished in MI compared with sham. Three of 5 MI mice developed hemodynamic instability at 20 μg/kg/min of dobutamine infusion. Of these 3, 2 mice subsequently died of cardiovascular collapse. Echocardiographic measurements at 7 wk were not included in Figure 2 because the method of anesthesia was different, which caused a significantly lower heart rate than under isoflurane anesthesia. Nevertheless, the measured echocardiographic parameters at 7 wks for both MI and sham were comparable from those obtained at 5 wks (data not shown).

### IWS changes in relation to dobutamine stimulation

**Figure 5:**
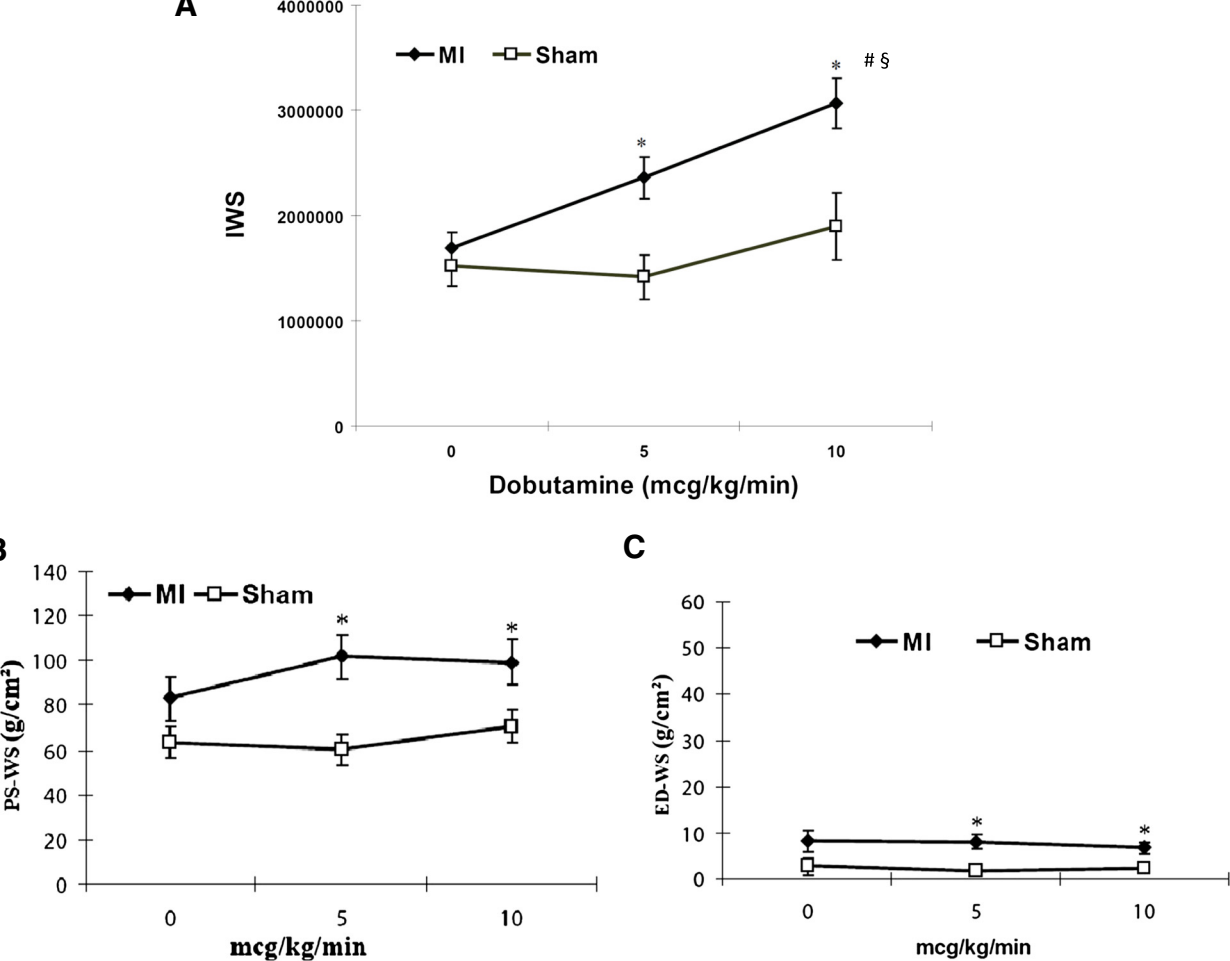
**Changes in IWS, peak systolic WS, and end diastolic WS with dobutamine stimulation. A**: IWS changes with incremental dobutamine stress. The IWS were comparable between MI (n = 5) and sham group (n = 5) at the baseline. However, IWS in MI hearts increased significantly with dobutamine stimulation, whereas there was no noticeable increase of IWS in the sham hearts. ms: millisecond. Changes in peak systolic WS **(B)** and end diastolic WS **(C)** with incremental dobutamine stress after small MI. Both peak systolic WS and end diastolic WS in MI hearts were significantly higher than those in sham hearts with dobutamine stimulation. * *p* < 0.05 vs. sham, # *p* < 0.05 vs. 0 mcg/kg/min, § < 0.05 vs. 5 mcg/kg/min.

IWS changes in both MI and sham groups, before and after dobutamine stimulation (5 and 10 μg/kg/min), are shown in Figure 5A. IWS was comparable between the two groups at the baseline. But with dobutamine stimulation, IWS was significantly increased in MI group, whereas IWS was relatively unchanged in sham group. Increased IWS implies increased external ventricular work load that is not absorbed by the intrinsic myocardial contractile reserve. The difference in myocardial reserve capacity in the two groups was reflected in IWS changes with dobutamine stimulation. Increased IWS by dobutamine stimulation in MI mice indicates increased ventricular workload in MI mice in a stressed condition.

We also measured peak systolic wall stress (PS-WS) and end diastolic wall stress (ED-WS) before and after dobutamine infusion in both groups (Figures 5B and C). At the resting condition, there was no significant difference in either PS-WS or ED-WS between MI and sham groups, although values in MI appear slightly higher than those of sham. However, with dobutamine stimulation, PS-WS in MI became significantly higher than that in sham at both 5 and 10 μg/kg/min infusion. This is because peak-systolic LV cavity size (LVIDs) in MI was higher than in sham and because peak-systolic LV wall thickness (LVPWs) in MI was lower than in sham, both of which contributed to higher PS-WS despite lower peak systolic LV pressure in MI mice than in sham. On the other hand, although ED-WS was persistently higher in MI than in sham, absolute values of ED-WS were far lower than those of PS-WS in both MI and sham hearts (approximately 10% of PS-WS). Thus, PS-WS, along with HR, is a predominant determinant for overall IWS in this small MI model.

### Relationship between IWS and the product of PS-WS and heart rate

**Figure 6:**
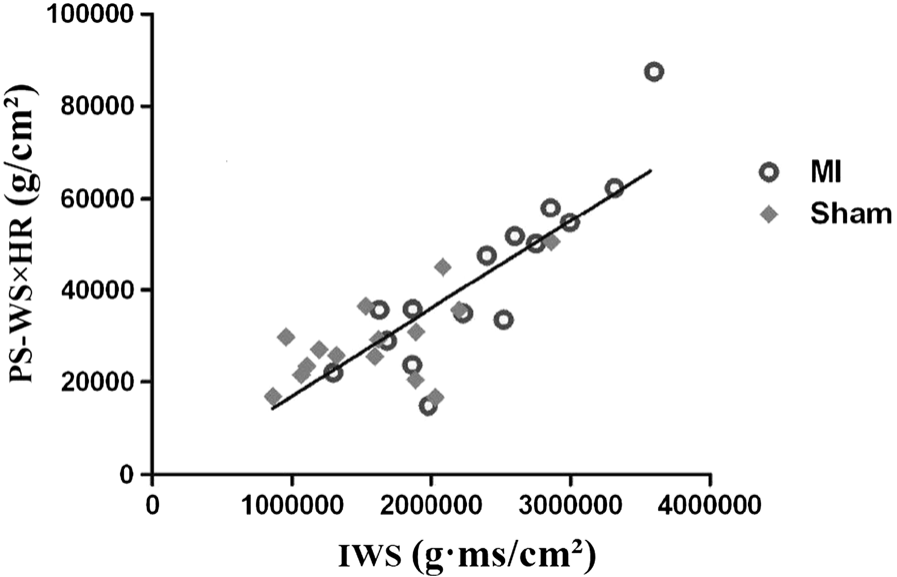
**Correlation between IWS and “IWS index (= PS-WS × HR)”.** There is an excellent correlation between IWS and peak systolic WS (PS-WS) × HR.

Increased IWS in dobutamine-stimulated condition in the MI group appeared to be primarily related to increased heart rate and higher PS-WS when compared with the sham group. Thus, we calculated the “IWS index” by multiplying PS-WS with heart rate as a marker for IWS, and examined whether IWS correlates well with calculated IWS index (= PS-WS **×** HR). Figure 6 shows that there is a good correlation between PS-WS **×** HR and IWS, both in sham-operated and MI hearts (*y* = 0.0191 *x* – 2155.4, R = 0.7003).

## Discussion

IWS measures total wall stress throughout the cardiac cycle over a unit time (= 1 min) including the effect of heart rate and inotropic state of the ventricular myocardium, whereas one-spot measurement of PS-WS and ED-WS only reflects maximum and minimum wall stress during a cardiac cycle, respectively. We hypothesized that increase in IWS indicates failure of myocardium to counteract increased ventricular workload. We have measured IWS in the mouse model in various physiological and pathological conditions to validate this hypothesis. Unchanged IWS observed in sham operated mice may imply that the contractile reserve of ventricular myocardium can absorb the increased cardiac output, whereas increased IWS after MI suggests that ventricular workloads exceeds intrinsic myocardial contractile reserve. Thus, we postulate that IWS is a reliable physiological marker in indicating a balance between external ventricular workload and intrinsic myocardial contractile reserve.

### IWS and myocardial reserve

“Wall stress theory” is an important concept in understanding the process of cardiac hypertrophy in response to increased hemodynamic loading [16]. When the LV myocardium encounters biomechanical overload, either pressure overload or volume overload, cardiac hypertrophy is naturally induced to normalize the wall stress so that myocardium can minimize the increase in myocardial oxygen demand; myocardial oxygen consumption depends mainly on systolic wall stress, heart rate, and contractility [8,17]. A question arises whether this hypertrophic response is a compensatory physiological adaptation to stabilize the wall stress or a pathological process leading to ventricular remodeling and heart failure. Physiological hypertrophy as seen in trained athletes reveals increased contractile reserve, whereas pathological hypertrophy shows a decrease in contractile reserve in addition to molecular expression of ventricular remodeling [18-20]. However, what regulates the transition from compensatory adaptation to maladaptive process is not well understood.

Systolic wall stress has been studied extensively as a clinical marker for myocardial reserve. Systolic wall stress reflects the major determinants of the degree of LV hypertrophy and plays a predominant role in LV function and myocardial energy balance [17]. It has been shown that increased systolic wall stress inversely correlates with systolic function and myocardial reserve in patients with chronic volume overload [9,10,21], chronic pressure overload [22,23], and dilated cardiomyopathy [24]. However, one-point measurement of systolic wall stress does not encompass the effect of heart rate and contractile status, the other critical factors that affect myocardial oxygen demand [11]. The idea of IWS has been proposed to incorporate wall stress throughout the cardiac cycle and reflects the effects of heart rate and contractile status.

Myocardial oxygen consumption is determined mainly by ventricular wall stress, heart rate and contractility [17], which are all incorporated in IWS measurement. Continuous measurement of LV wall stress was previously reported in humans [12,15] and dogs [11] with a similar method, but not in mice. By integrating the continuous WS over one minute, we estimated the balance between myocardial contractile reserve and total external ventricular workload and examined its trend in relation to inotropic stimulation in the mouse heart *in vivo*. In this study, we have proposed unchanged IWS as a marker of sufficient myocardial contractile reserve, since increased wall stress demands higher myocardial oxygen consumption. Indeed, systolic wall stress does not increase with strenuous isometric exercise in healthy young athletes [25]. Thus, we propose that increase in IWS indicates diminished myocardial contractile reserve.

### Small MI model as a unique model to study early phase of progressive ventricular remodeling

A complex series of protective and damaging events takes place after MI, resulting in increased ventricular workload [26]. Initial ventricular geometric change is considered as a primary compensatory response to counteract an abrupt loss of contractile tissue. In classical theories of wall stress, which rely on the law of Laplace, the mechanisms of progressive ventricular dilatation and functional deterioration of the LV are attributed to the increased wall stress that is not compensated by the intrinsic compensatory mechanisms [2,16]. Although this theory is obvious in advanced stage of heart failure, the subclinical ventricular remodeling following borderline cases such as following small MI with initial full compensatory response is not well explained.

We have shown that our small MI model induced concentric hypertrophy without LV dilatation as if initial myocardial damage was completely compensated (Figure 2) [5]. Although LV hypertrophy is induced initially to normalize the wall stress and to prevent ventricular dilatation, this hypertrophy is not altogether a physiological one because of decreased inotrophic and lusitropic reserve when stimulated with dobutamine (Figure 4) and because of simultaneous molecular and histological evidence of remodeling in the remote nonischemic LV myocardium (Figure 3). IWS and PS-WS become normalized in small MI at rest under anesthesia as a result of reactive hypertrophy accompanied by increased ANP and BNP mRNA level. Borderline maladaptive LVH is characterized by maintained LV performance at the expense of limited myocardial contractile reserve, and this abnormality can be unmasked by inotropic stimulation [18]. The trend of IWS at rest and with dobutamine stimulation suggests that MI mice were likely exposed to higher IWS during usual awake and active condition than sham-operated mice. In contrast, systolic wall stress in the pressure overload-induced LV hypertrophy showed a level comparable to that of sham both at rest and under stimulation by β1 adrenergic agonist, prenalterol, with comparable heart rate changes [27]. For this reason, IWS assessment by measuring cumulative WS in a unit time with and without inotropic stimuation should serve as a sensitive marker to assess whether induced LV hypertrophy is a compensatory physiological adaptation process or a pathological maladaptation process. Increased IWS that indicates imposed workload surpassing myocardial contractile reserve is likely to become a major driving factor in inducing progressive ventricular remodeling or initiating deleterious maladaptive processes after MI.

### IWS represents myocardial oxygen demand that can be estimated non-invasively

We have demonstrated a very good correlation between IWS and the product of PS-WS and HR (“IWS index”) in both MI and sham-operated hearts (Figure 6). This formula appears physiologically acceptable provided that ED-WS is sufficiently low compared with the PS-WS (approximately 10%, as is shown in Figures 4B and C). ES-WS was previously introduced as a useful tool for assessing myocardial loading status and myocardial oxygen consumption, but its measurement requires complicated preparation [28,29]. Because there is an excellent correlation between PS-WS and ES-WS, it has been demonstrated that ES-WS can be substituted by PS-WS [28], which can be easily obtained non-invasively [30]. ES-WS was previously determined as a useful marker to quantify LV afterload and contractility that can be simply and accurately measured non-invasively [15]. As myocardial oxygen consumption is mainly dependent upon systolic wall stress, contractility, and heart rate, it seems reasonable to propose that IWS and IWS index represent the status of myocardial contractile reserve.

### Study limitations

There are certain limitations in this study. First, wall stress measurement is reliable when there is an equal wall thickness with symmetrical structure. Obviously, with the creation of small MI, there is an asymmetry of LV myocardium in both structure and consistency (myocardium vs. scar tissue). However, the scar tissue is small and restricted to the LV apex (approximately 14% of entire LV myocardium [5]). In fact, most of LV wall was thickened after induction of this small experimental MI. Nevertheless, we acknowledge that this is our major limitation. Secondly, there is an individual variability in response to dobutamine stimulation even in sham mice. Although the average sham mice (n = 5) showed only a modest increase in HR, PS-WS, and IWS during dobutamine stimulation, one mouse presented in Figure 1 showed a notable increase in HR and PS-WS in response to dobutamine. Nevertheless, even with increased HR and PS-WS, the calculated IWS remained relatively unchanged in the sham-operated mice. Lastly, the reliability of IWS index is based upon the stipulation that ED-WS is significantly low compared with the systolic wall stress. Thus, IWS index may not be accurate in obvious volume overload cases and/or dilated hearts with LV dysfunction where ED-WS is significantly higher than that in normal condition. Of note, ED-WS in human is higher than that in mice in relation to PS-WS, probably around 15 to 20% of PS-WS [12].

### Clinical implications

IWS can be estimated by obtaining IWS index, which is calculated non-invasively by simultaneous M-mode echocardiogram and cuff blood pressure measurement, i.e., PS-WS instead of ES-WS and heart rate. This will provide a sensitive way to detect subclinical borderline failing myocardium in which the decline in LV myocardial contractile reserve precedes apparent LV dysfunction. This method may be clinically useful to address LV myocardial reserve in those patients who are not amenable to perform on exercise stress test, such as immediate post-operative patients under mechanical ventilation, critically ill patients with questionable LV dysfunction, and patients with primary muscular disorders and general muscular weakness (i.e., Duchenne muscular dystrophy).

## Competing interests

The authors declare that they have no competing interests.

## Authors’ contributions

HD prepared and participated in all animal studies, completed histology and molecular studies, critically analyzed the data, performed statistic analysis, and completed all figures. HR assisted in all aspects of the animal experiments and independently performed echocardiography. EG performed the surgical procedures for experimental MI and invasive cardiac catheterization. RAE and SGG critically read the manuscript and helped to revise the text. TT originally created the concept of IWS, designed the experimental plans, supervised all laboratory activities, assessed the data with co-authors, and drafted and revised the manuscript. All authors read and approved the final manuscript.

## References

[B1] Mann DL Mechanisms and models in heart failure: a combinatorial approach Circulation 1999 100 999 1008 10.1161/01.CIR.100.9.999 10468532

[B2] Sutton MG Sharpe N Left ventricular remodeling after myocardial infarction: Pathophysiology and therapy Circulation 2000 101 2981 2988 10.1161/01.CIR.101.25.2981 10869273

[B3] Colucci WSBE Braunwald E, Zipes D, Libby P Pathophysiology of heart failure Heart disease 2001 6 Philadelphia: Saunders 503 533

[B4] Opie L Opie L Ventricular function Heart physiology 2004 4 Philadelphia, Baltimore, New York, London, Buenos Aires, Hong Kong, Sydney and Tokyo: Lippincott Williams & Wilkins 351 401

[B5] Tsuda T Gao E Evangelisti L Markova D Ma X Chu ML Post-ischemic myocardial fibrosis occurs independent of hemodynamic changes Cardiovasc Res 2003 59 926 933 10.1016/S0008-6363(03)00519-4 14553832

[B6] Barbone A Oz MC Burkhoff D Holmes JW Normalized diastolic properties after left ventricular assist result from reverse remodeling of chamber geometry Circulation 2001 104 I229 I232 11568061 10.1161/hc37t1.094914

[B7] Cheng A Nguyen TC Malinowski M Langer F Liang D Daughters GT Ingels NB Jr Miller DC Passive ventricular constraint prevents transmural shear strain progression in left ventricle remodeling Circulation 2006 114 I79 I86 16820650 10.1161/CIRCULATIONAHA.105.001578

[B8] Strauer BE Beer K Heitlinger K Hofling B Left ventricular systolic wall stress as a primary determinant of myocardial oxygen consumption: Comparative studies in patients with normal left ventricular function, with pressure and volume overload and with coronary heart disease Basic Res Cardiol 1977 72 306 313 10.1007/BF01906378 140677

[B9] Osbakken M Bove AA Spann JF Left ventricular function in chronic aortic regurgitation with reference to end-systolic pressure, volume and stress relations Am J Cardiol 1981 47 193 198 10.1016/0002-9149(81)90383-0 6451165

[B10] Shen WF Fletcher PJ Roubin GS Harris PJ Kelly DT Relation between left ventricular functional reserve during exercise and resting systolic loading conditions in chronic aortic regurgitation Am J Cardiol 1986 58 757 761 10.1016/0002-9149(86)90351-6 3766416

[B11] Colin P Ghaleh B Monnet X Su J Hittinger L Giudicelli JF Berdeaux A Contributions of heart rate and contractility to myocardial oxygen balance during exercise Am J Physiol Heart Circ Physiol 2003 284 H676 H682 12399255 10.1152/ajpheart.00564.2002

[B12] Grossman W Jones D McLaurin LP Wall stress and patterns of hypertrophy in the human left ventricle J Clin Invest 1975 56 56 64 10.1172/JCI108079 124746 PMC436555

[B13] Gao E Lei YH Shang X Huang ZM Zuo L Boucher M Fan Q Chuprun JK Ma XL Koch WJ A novel and efficient model of coronary artery ligation and myocardial infarction in the mouse Circ Res 2010 107 1445 1453 10.1161/CIRCRESAHA.110.223925 20966393 PMC3005817

[B14] Kamphoven JH Stubenitsky R Reuser AJ Van Der Ploeg AT Verdouw PD Duncker DJ Cardiac remodeling and contractile function in acid alpha-glucosidase knockout mice Physiol Genomics 2001 5 171 179 11328962 10.1152/physiolgenomics.2001.5.4.171

[B15] Reichek N Wilson J St John Sutton M Plappert TA Goldberg S Hirshfeld JW Noninvasive determination of left ventricular end-systolic stress: Validation of the method and initial application Circulation 1982 65 99 108 10.1161/01.CIR.65.1.99 7053293

[B16] Grossman W Cardiac hypertrophy: Useful adaptation or pathologic process? Am J Med 1980 69 576 584 10.1016/0002-9343(80)90471-4 6448546

[B17] Strauer BE Left ventricular dynamics, energetics and coronary hemodynamics in hypertrophic heart disease Eur Heart J 1983 4 Suppl A 137 142 10.1093/eurheartj/4.suppl_A.137 6220892

[B18] Fontanet HL Perez JE Davila-Roman VG Diminished contractile reserve in patients with left ventricular hypertrophy and increased end-systolic stress during dobutamine stress echocardiography Am J Cardiol 1996 78 1029 1035 8916483

[B19] Force T Michael A Kilter H Haq S Stretch-activated pathways and left ventricular remodeling J Card Fail 2002 8 S351 S358 10.1054/jcaf.2002.129272 12555145

[B20] Weber KT Clark WA Janicki JS Shroff SG Physiologic versus pathologic hypertrophy and the pressure-overloaded myocardium J Cardiovasc Pharmacol 1987 10 Suppl 6 S37 S50 2485029

[B21] Borow KM Green LH Mann T Sloss LJ Braunwald E Collins JJ Cohn L Grossman W End-systolic volume as a predictor of postoperative left ventricular performance in volume overload from valvular regurgitation Am J Med 1980 68 655 663 10.1016/0002-9343(80)90251-X 7377221

[B22] Krayenbuehl HP Hess OM Ritter M Monrad ES Hoppeler H Left ventricular systolic function in aortic stenosis Eur Heart J 1988 9 Suppl E 19 23 10.1093/eurheartj/9.suppl_E.19 2969811

[B23] Yuda S Khoury V Marwick TH Influence of wall stress and left ventricular geometry on the accuracy of dobutamine stress echocardiography J Am Coll Cardiol 2002 40 1311 1319 10.1016/S0735-1097(02)02105-8 12383580

[B24] Paraskevaidis IA Tsiapras DP Adamopoulos S Kremastinos DT Assessment of the functional status of heart failure in non ischemic dilated cardiomyopathy: an echo-dobutamine study Cardiovasc Res 1999 43 58 66 10.1016/S0008-6363(98)00345-9 10536690

[B25] Haykowsky M Taylor D Teo K Quinney A Humen D Left ventricular wall stress during leg-press exercise performed with a brief valsalva maneuver Chest 2001 119 150 154 10.1378/chest.119.1.150 11157597

[B26] Opie LHCP Gersh B Pfeffer MA Controversies in ventricular remodeling Lancet 2006 367 356 367 10.1016/S0140-6736(06)68074-4 16443044

[B27] Fujii AM Vatner SF Serur J Als A Mirsky I Mechanical and inotropic reserve in conscious dogs with left ventricular hypertrophy Am J Physiol 1986 251 H815 H823 2945443 10.1152/ajpheart.1986.251.4.H815

[B28] Colan SD Borow KM MacPherson D Sanders SP Use of the indirect axillary pulse tracing for noninvasive determination of ejection time, upstroke time, and left ventricular wall stress throughout ejection in infants and young children Am J Cardiol 1984 53 1154 1158 10.1016/0002-9149(84)90653-2 6702695

[B29] Colan SD Borow KM Neumann A Effects of loading conditions and contractile state (methoxamine and dobutamine) on left ventricular early diastolic function in normal subjects Am J Cardiol 1985 55 790 796 10.1016/0002-9149(85)90158-4 2579537

[B30] Borow KM Green LH Grossman W Braunwald E Left ventricular end-systolic stress-shortening and stress-length relations in human. Normal values and sensitivity to inotropic state Am J Cardiol 1982 50 1301 1308 10.1016/0002-9149(82)90467-2 7148706

